# The added diagnostic value of complementary gadoxetic acid-enhanced MRI to ^18^F-DOPA-PET/CT for liver staging in medullary thyroid carcinoma

**DOI:** 10.1186/s40644-019-0263-z

**Published:** 2019-11-14

**Authors:** Daniel Puhr-Westerheide, Clemens C. Cyran, Josef Sargsyan-Bergmann, Andrei Todica, Franz-Josef Gildehaus, Wolfgang G. Kunz, Robert Stahl, Christine Spitzweg, Jens Ricke, Philipp M. Kazmierczak

**Affiliations:** 1 Department of Radiology, University Hospital, LMU Munich, Marchioninistraße 15, 81377 Munich, Germany; 2 Department of Nuclear Medicine, University Hospital, LMU Munich, Marchioninistraße 15, 81377 Munich, Germany; 3 Department of Neuroradiology, University Hospital, LMU Munich, Marchioninistraße 15, 81377 Munich, Germany; 4 Department of Internal Medicine IV, University Hospital, LMU Munich, Marchioninistraße 15, 81377 Munich, Germany

**Keywords:** Magnetic resonance imaging, Positron emission tomography computed tomography, Neoplasm metastasis, Liver, Contrast media

## Abstract

**Background:**

A high proportion of patients with advanced stages of medullary thyroid carcinoma (MTC) present with liver metastasis metastases. The aim of our study was to investigate the added diagnostic value of complementary gadoxetic acid-enhanced MRI to ^18^F-DOPA-PET/CT for liver staging in MTC.

**Methods:**

Thirty-six patients (14 female, median age 55 years) with histologically confirmed MTC undergoing gadoxetic acid-enhanced liver MRI within 1 month of matching contrast-enhanced ^18^F-DOPA-PET/CT between 2010 and 2016 were selected for this IRB-approved retrospective study. ^18^F-DOPA-PET/CT and multiparametric MRI data sets were read consecutively and liver lesions were categorised on a 5-point Likert scale (1–definitely benign; 2–probably benign; 3–intermediate risk for metastasis; 4–probably metastasis; 5–definitely metastasis). It was noted if gadoxetic acid-enhanced MRI detected additional, ^18^F-DOPA-PET/CT-occult metastases (category 5) or if gadoxetic acid-enhanced MRI allowed for a definite classification (categories 1 and 5) of lesions for which ^18^F-DOPA-PET/CT remained inconclusive (categories 2–4). Follow-up PET/CT and MRI examinations were used as a reference standard.

**Results:**

A total of 207 liver lesions (^18^F-DOPA-PET/CT 149, MRI 207; 152 metastases, 37 benign cysts, 18 hemangiomas) were analysed. Fifty-eight additional lesions were detected by MRI, of which 54 were metastases (median diameter 0.5 cm [interquartile range 0.4–0.7 cm]) occult on ^18^F-DOPA-PET/CT. MRI allowed for a definite lesion classification (categories 1 and 5) in 92% (190/207) whereas ^18^F-DOPA-PET/CT allowed for a definite lesion classification in 76% (113/149). MRI lead to a change in lesion categorisation in 14% (21/149).

**Conclusion:**

Gadoxetic acid-enhanced MRI allows for a more precise liver staging in MTC patients compared to ^18^F-DOPA-PET/CT alone, particularly for ^18^F-DOPA-negative metastases and lesions < 1 cm.

## Key points

- Combining gadoxetic acid-enhanced MRI and^18^F-DOPA-PET/CT optimises liver staging in MTC patients.
- Gadoxetic acid-enhanced MRI is particularly helpful for the detection and characterisation of small (< 1 cm) liver lesions.

## Background

Medullary thyroid carcinoma (MTC) accounts for 1–2% of all thyroid malignancies and causes up to 13% of all thyroid disease-related deaths [1, 2]. Sporadic occurrence encompasses 75% of all MTC cases while the remaining 25% are associated with hereditary tumour syndromes (e.g., multiple endocrine neoplasia (MEN) 2A and 2B). MTC-related lethality is mostly due to distant metastases and the median 10-year survival upon advanced stages of the disease is reported to be 10% [3, 4]. 13 to 15% of patients present with distant metastases at the time of diagnosis [5]. Medullary thyroid carcinoma is a malignant neuroendocrine tumour with the capability to take up amine precursors, such as dopamine, for decarboxylation (Amine Precursor Uptake and Decarboxylation system), thereby allowing the use of ^18^F-DOPA as a radiotracer for the detection of metastases. Particularly ^18^F-DOPA-PET/CT has been recognized as a highly sensitive and specific imaging modality for the detection of metastatic MTC [6–10].

The liver is the most frequently affected organ, with liver metastases in 45% of patients with advanced MTC [1]. However, liver staging in MTC remains challenging, as small metastases may remain ^18^F-DOPA-negative. A timely and comprehensive liver staging is of major importance to evaluate potential treatment options with a growing oncological toolbox including local ablative treatment, surgical, or systemic therapy [11–19]. Recent studies have shown that contrast-enhanced liver magnetic resonance imaging (MRI) is best suited for the detection of malignant liver lesions, particularly small metastases < 1.0 cm [20–24]. Gadoxetic acid is a contrast medium which specifically distributes into hepatocytes and the biliary tract system in a late, hepatobiliary phase. This allows for a differentiation of hepatocytes from neoplastic cells, which do not show a gadoxetic acid storage, thereby rendering gadoxetic acid a valuable contrast agent in patients with suspected hepatocellular carcinoma [25–27] or suspected liver metastases [28–30]. However, the value of contrast-enhanced liver MRI in patients with metastatic MTC was not yet investigated and the current guidelines do not recommend gadoxetic acid-enhanced MRI as routine liver staging in MTC. Therefore, the aim of the present study was to investigate the added diagnostic value of complementary gadoxetic acid-enhanced liver MRI to ^18^F-DOPA-PET/CT for liver staging in MTC.

We hypothesised that gadoxetic acid-enhanced liver MRI

- provides a higher liver metastasis detection rate than ^18^F-DOPA-PET/CT, and
- allows for a definite liver lesion classification when ^18^F-DOPA-PET/CT remains inconclusive

in patients with histologically confirmed MTC.

## Methods

This retrospective study was approved by the Institutional Review Board and the requirement for informed consent was waived. Written informed consent for the diagnostic ^18^F-DOPA PET/CT scan and the contrast-enhanced MRI scan was obtained from all patients prior to the examination.

### Study population

**Fig. 1:**
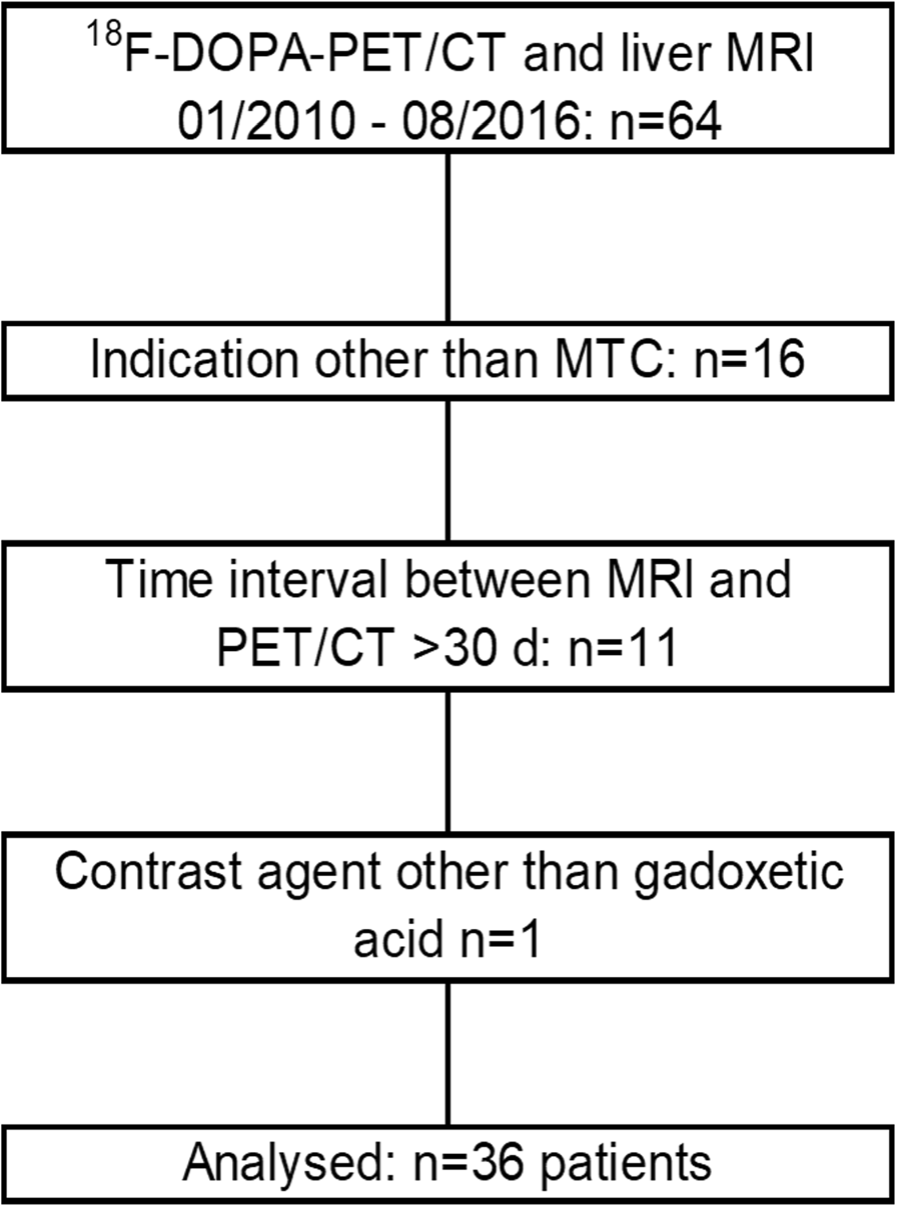
Patient inclusion flowchart

Patients with histologically confirmed MTC who underwent gadoxetic acid-enhanced liver MRI and ^18^F-DOPA-PET/CT for whole-body tumour staging within 30 days between 2010 and 2016 were included in the analysis. Detailed inclusion and exclusion criteria are provided in Table 1, (Fig. 1) visualises the process of patient selection.

**Table 1:** Inclusion and exclusion criteria

Inclusion criteria	Exclusion criteria
Histologically confirmed MTC (surgical thyroidectomy or fine needle aspiration)	Unenhanced acquisition of the MRI scan (due to contraindications to gadolinium-based contrast agents, such as acute or chronic renal failure with estimated glomerular filtration rate < 30 mL/min or known hypersensitivity)
Whole-body ^18^F-DOPA PET/CT between 2010 and 2016	MRI contrast agent other than gadoxetic acid
Matching gadoxetic acid-enhanced liver MRI within 30 days	Unenhanced CT (due to contraindications for iodinated contrast agents, such as manifest hyperthyroidism, acute or chronic renal failure with estimated glomerular filtration rate < 30 mL/min or known hypersensitivity)
	Missing or inconclusive histological results confirming the diagnosis of MTC

### PET/CT protocol

A commercially available ^18^F-DOPA was used (IASOdopa®, IASON GmbH). A Biograph 64 TruePoint PET/CT scanner (Siemens Healthineers) was used for whole-body PET/CT imaging in three-dimensional mode (3 min per bed position). Patients were asked to empty their bladder prior to the examination. Furosemide (20 mg) and ^18^F-DOPA (median 255 MBq, range 236–272 MBq) were consecutively administered intravenously and positron emission scans were initiated approximately 60 min after injection. A diagnostic CT scan covering the neck, chest, abdomen, and pelvis was acquired with automatic exposure control using tube current modulation (CARE Dose 4D; Siemens Healthineers; scan parameters: 120 kV, 100–190 mAs, collimation 2 × 5 mm, pitch 1.5) in portal venous phase (individually calculated according to the formula: Delay [s] = (VolumeContrast Agent [mL] + Volume Saline [mL])/2.5 mL/s) with automated intravenous injection [2.5 mL/s] of an iodine-based contrast agent (Ultravist 300™; Bayer Healthcare; 1.5 mL/kg body weight) and a saline chaser of 100 mL. CT datasets were used for PET attenuation correction.

### MRI protocol

Multiparametric liver MRI was performed on a 1.5 Tesla scanner (MAGNETOM Avanto/Aera, Siemens Healthineers) using an 18-channel body array. Contrast-enhanced images were acquired using gadoxetic acid (Primovist/ Eovist, Bayer Vital GmbH; weight-adapted dose according to the manufacturer’s instructions). The hepatobiliary phase was acquired 20 min after intravenous gadoxetic acid injection. The multiphase multiparametric liver protocol included the following sequences: T1-weighted three-dimensional gradient echo (GRE) fat saturation sequences in unenhanced, arterial, portal venous, late dynamic, and hepatobiliary phase; T2-weighted single shot turbo-spin sequences (half-Fourier acquisition single-shot turbo spin-echo [HASTE]); T2-weighted turbo-spin-echo (TSE) and diffusion weighted imaging (DWI, b-values 50, 400 and 800). Details can be obtained from Table 2.

**Table 2:** Sequence parameters of the comprehensive MRI protocol

Sequence	TR, ms	TE, ms	FOV, mm	FOV phase	Flip angle	B-value, s/mm^2^	Respiratory control
T1w in-phase	110	4.76	360	87.5%	70°	n/a	breath-hold
T1w out-of-phase	110	2.50	360	87.5%	70°	n/a	breath-hold
T1w 3D GRE fs unenhanced, arterial phase, portal venous phase, late dynamic phase, and hepatobiliary phase	3.35	1.19	400	87.5%	15°	n/a	breath-hold
T2w HASTE	800	54.00	360	88.8%	180°	n/a	trigger
T2w turbo-spin-echo	2860	107.00	360	87.5%	180°	n/a	breath-hold
Diffusion weighted imaging	2800	80.00	400	75.0%	n/a	50	breath-hold
Diffusion weighted imaging	2800	80.00	400	75.0%	n/a	400	breath-hold
Diffusion weighted imaging	2800	80.00	400	75.0%	n/a	800	breath-hold

*GRE* gradient echo, *fs* fat sat, *HASTE* half fourier acquisition single shot turbo spin echo, *TR* time to repetiton, *TE* time to echo, *FOV* field of view

### Blinded reading

First, two blinded radiologists (C. C. C. and P. M. K., 10 and 6 years of experience in oncological whole-body imaging and MRI reading; C. C. C. is additionally certified in diagnostic nuclear medicine) independently evaluated the co-registered contrast-enhanced CT and PET datasets side by side on a clinical workstation using dedicated image postprocessing software (syngo.via; Siemens Healthineers). Second, the readers analysed the liver MRI datasets on a clinical workstation (first reading: comprehensive, multi-sequence MRI protocol including all sequences; second reading: DWI). For each modality, any detectable liver lesions were systematically classified on a 5-point Likert scale applying the following lesion classification: 1 – definitely benign; 2 – probably benign; 3 – intermediate risk for malignancy; 4 – probably malignant; 5 – definitely malignant.

The following malignancy criteria were applied:

1. ^18^F-DOPA-avidity
2. Hyperenhancement on multiphasic MRI
3. Hyperenhancement on ^18^F-DOPA-PET/CT
4. MRI: Wash-out on the late dynamic phase
5. MRI: Presence of a capsule or pseudocapsule
6. Restricted diffusion as supporting co-feature [28, 31]

Target parameters were a change in lesion category based on gadoxetic acid-enhanced MRI and the detection of ^18^F-DOPA PET/CT-occult metastases. A consensus reading was performed in case of divergent results. Combined ^18^F-DOPA-PET/CT and MRI follow-up scans were used as reference standard.

### Clinical data analysis

The following clinical parameters were documented for each patient: age and sex, presence of hereditary tumour syndromes, lymph node metastases, distant metastases other than hepatic, serum calcitonin concentration, and carcinoembryonic antigen (CEA) levels at the time of the scan. In addition, the interval between the ^18^F-DOPA-PET/CT and the gadoxetic acid-enhanced MRI scan was noted.

### Patient characteristics

In total, 36 consecutive patients (14 female; median age 55 years, interquartile range: 43–67 years) were included in the retrospective analysis. The study population included mostly patients with sporadic MTC (31/36, 86%), three patients had underlying MEN 2a, two patients had other mutations predisposing for MTC. In the majority of cases, primary tumour surgery (thyroidectomy with lymph node dissection) with confirmation of the diagnosis by postoperative histopathology was performed in an external hospital (33/36, 92%). 86% of patients presented with lymph node metastases, 44% with liver metastases, and 42% with distant metastases other than hepatic. The median interval between the initial ^18^F-DOPA PET/CT scan and the complementary gadoxetic acid-enhanced liver MRI was 3 days (interquartile range 0 to 12 days). Table 3 shows the patient characteristics for patients included in the retrospective analysis.

**Table 3:** Patient characteristics

Patient data	
Number of patients	36
Age (years)	55 (43–67)
Female	14 (39%)
Calcitonin level (pg/mL)	1377 (279–2850)
CEA level (pg/mL)	19 (5–125)
Liver metastases	16 (44%)
Lymph node metastases	31 (86%)
Pulmonary metastases	11 (31%)
Bone metastases	7 (19%)
Soft tissue metastases	1 (3%)
Hereditary MTC	5 (14%)

Values presented are count (percentage) for categorical and median (interquartile range) for ordinal or continuous variables

*CEA* carcinoembryonic antigen, *MTC* medullary thyroid carcinoma

### Statistical analysis

The statistical analysis was performed using SPSS 21 for Windows (IBM Corp.). The Wilcoxon signed-rank test for related groups was used to detect differences in the number of detected lesions or in lesion categorisation between ^18^F-DOPA PET/CT scans and gadoxetic acid-enhanced MRI scans. Statistical significance was assumed for *p*-values < 0.05.

## Results

**Fig. 2:**
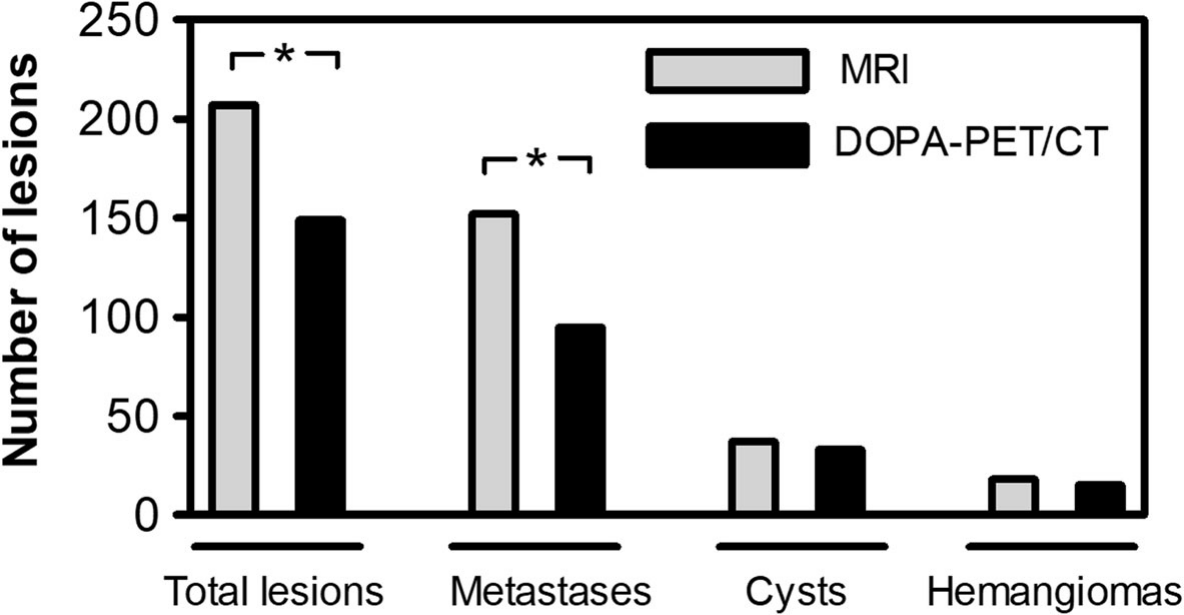
Lesion detection on MRI compared to ^18^F-DOPA-PET/CT. Significantly more lesions were detected on MRI (* *p* < 0.001). MRI revealed significantly more metastases compared to ^18^F-DOPA-PET/CT (* *p* = 0.002)

**Fig. 3:**
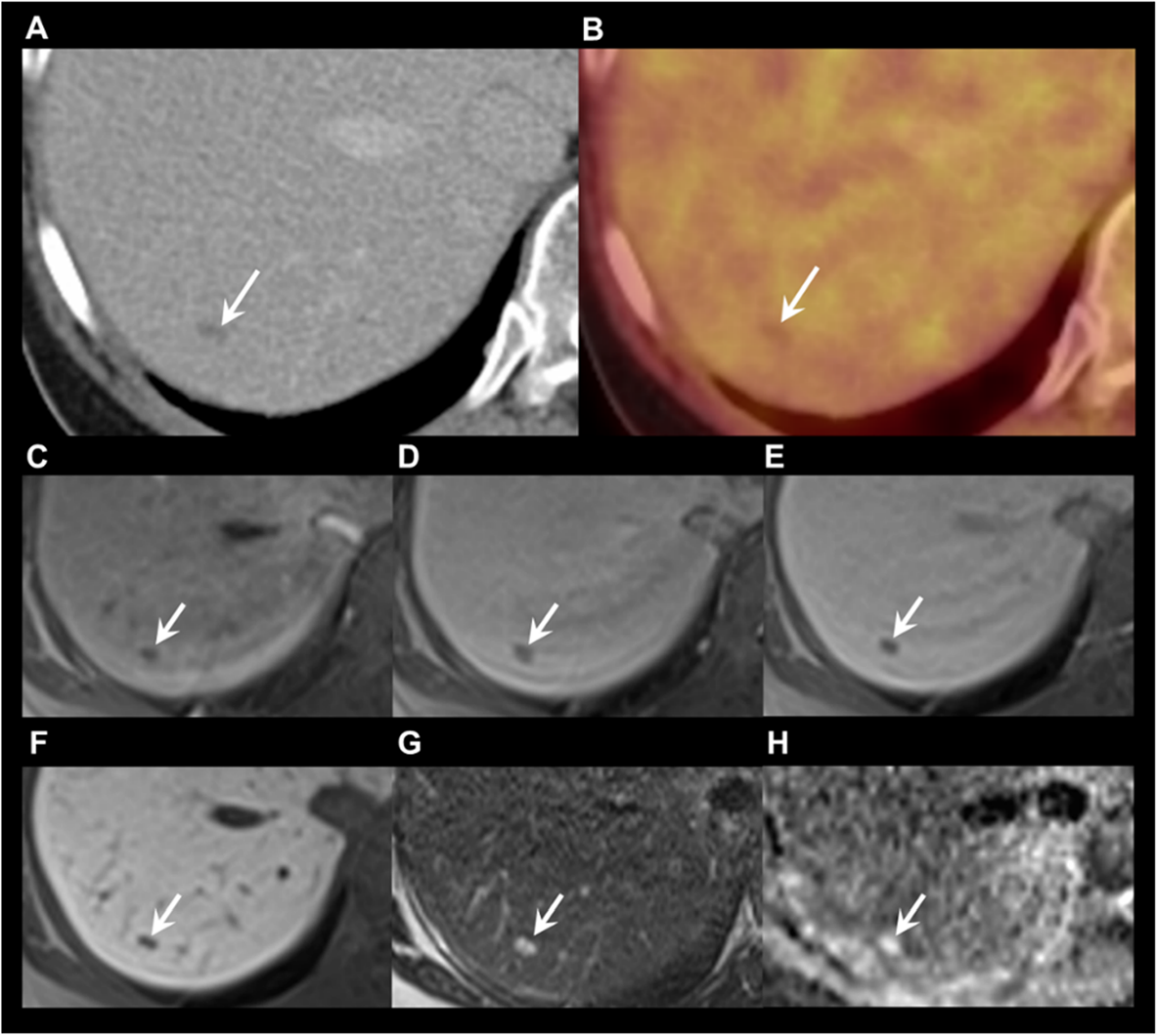
Lesion in segment VII with a change in categorization from category 2 on ^18^F-DOPA-PET/CT to category 1 through additional features on gadoxetic acid-enhanced MRI. **a** Contrast enhanced CT. **b** ^18^F-DOPA-PET/CT (fusion image). **c**, **d**, **e**, **f** T1 GRE fs with arterial, portal venous, late dynamic, and hepatobiliary phase. **g** T2. H: DWI, b800. ^18^F-DOPA-PET/CT **a** and **b** shows a small hypodense lesion (arrow) without ^18^F-DOPA uptake read as category 2 (probably benign). As MRI showed no contrast enhancement (**c**-**e**), no diffusion restriction but T2 shine through in the ADC map (**h**), and it appeared homogenously hyperintense on T2 (**g**), it was categorised as a benign cyst (category 1 – definitely benign)

**Fig. 4:**
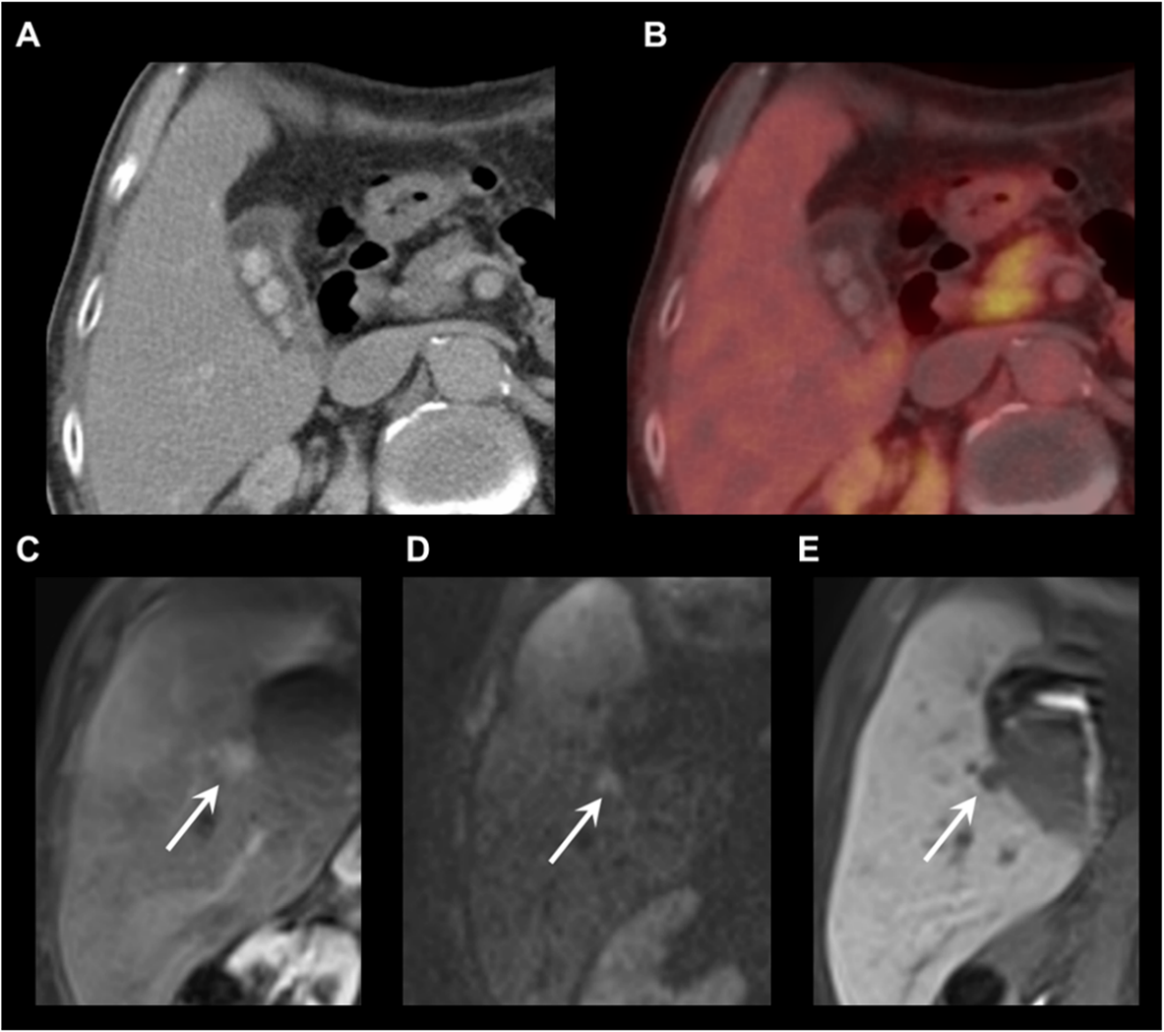
Detection of an ^18^F-DOPA-PET/CT occult liver metastasis on gadoxetic acid-enhanced MRI. **a** Contrast-enhanced CT. **b** ^18^F-DOPA-PET/CT (fusion image). **c** T1 GRE fs, arterial phase. **d** DWI, b800. **e** T1 GRE fs, hepatobiliary phase. ^18^F-DOPA-PET/CT shows no liver lesion. MRI reveals an ^18^F-DOPA-PET/CT-occult, hypervascular metastasis in liver segment V (C-E, arrows)

**Fig. 5:**
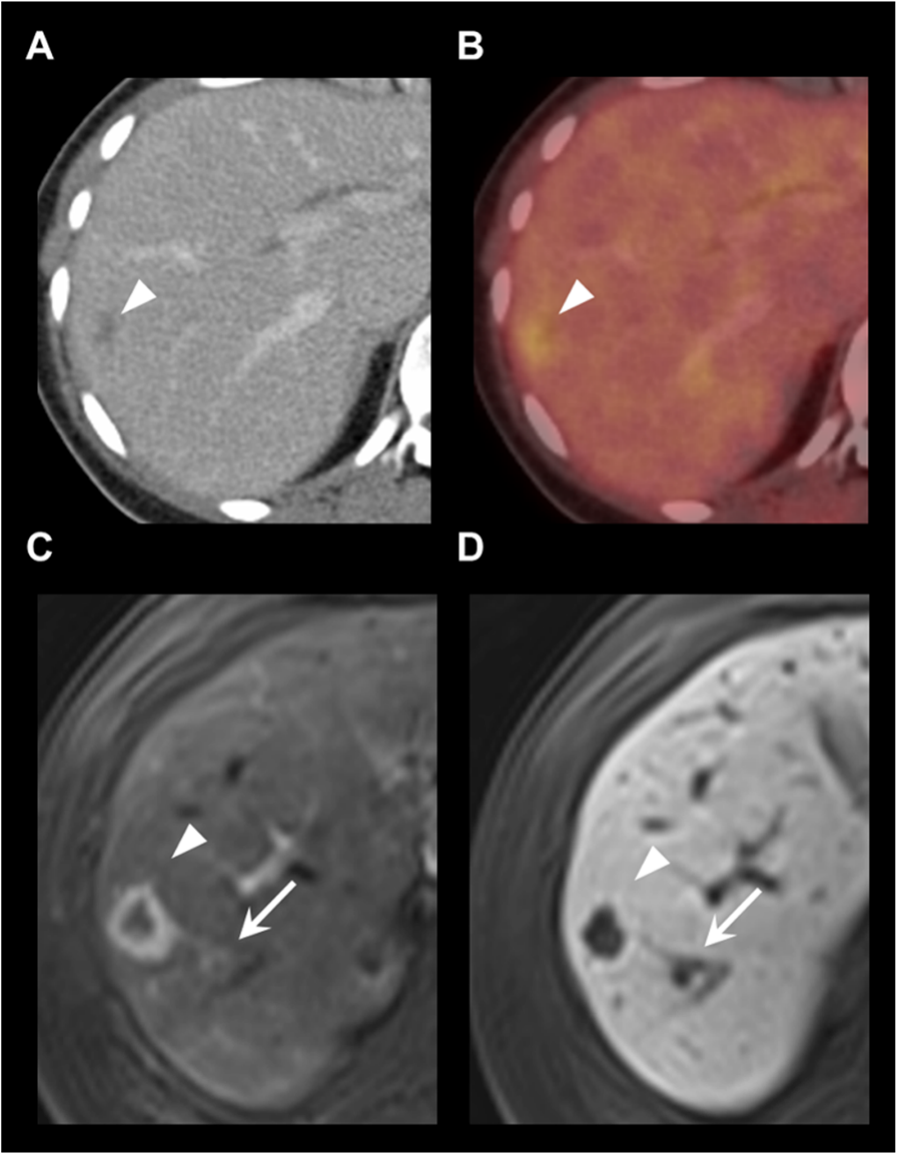
Detection of an additional, ^18^F-DOPA-PET/CT-occult metastasis on gadoxetic acid-enhanced MRI. **a** Contrast-enhanced CT. **b** ^18^F-DOPA-PET/CT (fusion image). **c** T1 GRE fs, arterial phase. **d** T1 GRE fs, hepatobiliary phase. Hypodense lesion on contrast-enhanced CT with significant ^18^F-DOPA uptake (**a**-**b**, arrowhead). MRI reveals an additional hypervascular, ^18^F-DOPA-PET/CT-occult metastasis in close proximity (**c**-**d**, arrow)

**Fig. 6:**
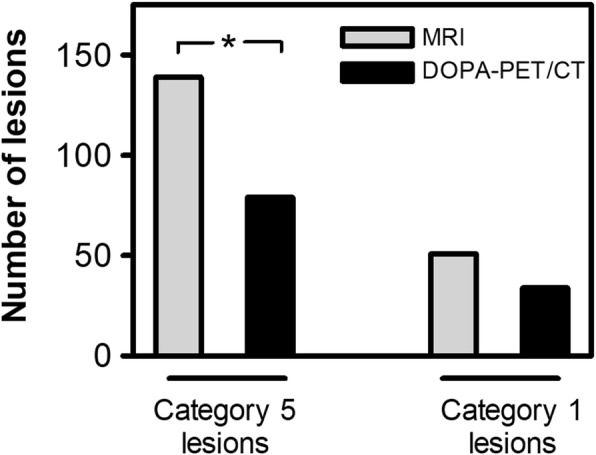
Detection of category 5 and category 1 lesions on gadoxetic acid-enhanced MRI and ^18^F-DOPA-PET/CT. Gadoxetic acid-enhanced MRI allows for definite lesion categorisation at a significantly higher rate. * *p* = 0.001

In total, 207 liver lesions were detected and classified in 36 patients. The number of lesions detected with ^18^F-DOPA-PET/CT and gadoxetic acid-enhanced MRI is shown in Table 4. ^18^F-DOPA-PET/CT detected 149 lesions (72% of all lesions, 149/207). Six lesions (3%, 6/207) remained category 3 lesions based on the ^18^F-DOPA-PET/CT and could neither be classified as benign nor as malignant. In contrast, MRI detected 207 lesions. All lesions could be characterized as benign (category 1 or 2) or malignant (category 4 or 5). Differences between the number of detected lesions with ^18^F-DOPA-PET/CT and gadoxetic acid-enhanced MRI can be seen in (Fig. 2). MRI detected significantly more lesions than ^18^F-DOPA-PET/CT (*p* < 0.001) and significantly more lesions could be categorised as metastases (category 4 and 5, *p* = 0.001). MRI allowed for a definite lesion classification (category 1 or 5) in 92% of all lesions (190/207) with 51 category 1 lesions (25%, 51/207) and 139 category 5 lesions (67%, 139/207). In contrast, ^18^F-DOPA-PET/CT was not as conclusive in lesion categorisation as gadoxetic acid-enhanced MRI. Of the 149 lesions detected by ^18^F-DOPA-PET/CT, 76% could be classified as category 1 or 5 lesions (113/149). Figures 3, 4, and 5 show exemplary cases in which gadoxetic acid-enhanced MRI provided added diagnostic value. MRI detected significantly more category 5 lesions than ^18^F-DOPA-PET/CT (*p* < 0.001) (Fig. 6).

**Table 4:** Absolute liver lesion counts for ^18^F-DOPA-PET/CT and gadoxetic acid-enhanced MR

	Number of lesions		
	^18^F-DOPA-PET/CT	Gadoxetic acid-enhanced MRI	Additional lesions detected by MRI
All lesion categories	149	207	58
Metastases (category 4, 5)	95	152	54
Cysts	33	37	1
Hemangiomas	15	18	3
Category 1	34	51	
Category 2	14	4	
Category 3	6	0	
Category 4	16	13	
Category 5	79	139	
	Change in lesion category		
	^18^F-DOPA-PET/CT-related	Gadoxetic-acid enhanced MRI-related	
Category 2 to 1	0	10	
Category 3 to 1	0	3	
Category 3 to 5	0	3	
Category 4 to 5	0	5

Overall, gadoxetic acid-enhanced MRI detected 58 additional lesions. 54 (93%) of these ^18^F-DOPA-PET/CT-occult lesions were categorized as metastases. The additionally detected metastases were small (0.5 cm, interquartile range 0.4–0.7 cm).

On MRI, no definite lesion characterisation (categories 2–4) was rare (8% of all lesions, 17/207) whereas ^18^F-DOPA-PET/CT did not allow for a definite lesion categorisation in 36 lesions (24% of all lesions detected by ^18^F-DOPA-PET/CT, 36/149). Changes in lesion categorisation based on the MRI scan can be obtained from Table 4. Of note, all liver metastases could successfully be diagnosed using the multiphase contrast-enhanced sequences, the T2-weighted sequences, and the DWI. The hepatobiliary phase did not lead to the detection of additional metastases but added significantly to the level of confidence in lesion characterisation.

**Fig. 7:**
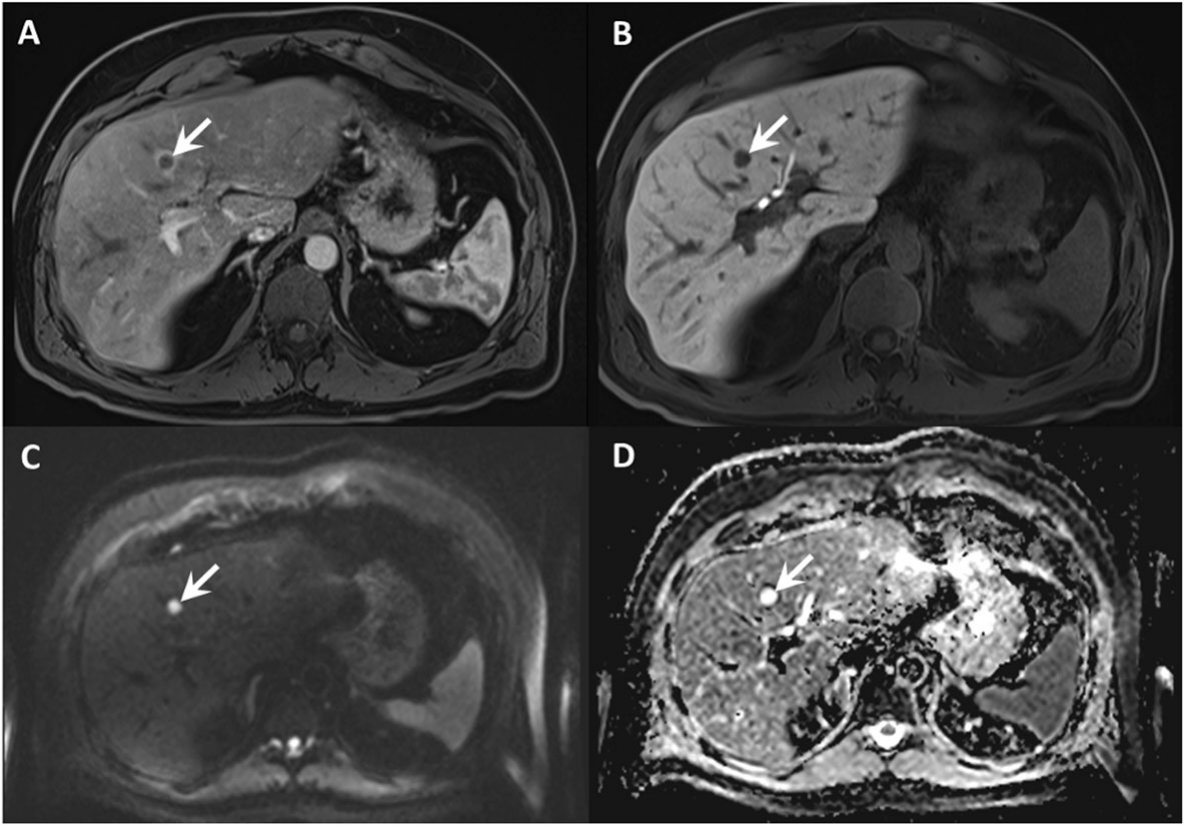
Lesion detection on contrast enhanced images compared to DWI and ADC maps. **a**, **b** T1 GRE fs with arterial and hepatobiliary phase. **c**, **d** DWI, b800 and ADC map. Contrast enhanced T1 images show a centrally hypointense lesion (arrow) with a hyperenhancing rim in the arterial phase and no contrast uptake in the hepatobiliary phase, indicating a partially cystic metastasis (**a** and **b**). Note the T2-shine through on DWI due to its cystic character (**c** and **d**)

Of note, one patient in the investigated cohort did not show any metastatic lesions on ^18^F-DOPA-PET/CT but one metastasis (category 5 lesion) on multiparametric liver MRI. Of the 152 metastases detected on multiparametric MRI, 84 (55% of all detected metastases) demonstrated a diffusion restriction. However, a significant proportion (56/152, 37%) of the detected metastases demonstrated a brisk arterial rim enhancement and a cystic centre, resulting in T2-shine through (Fig. 7). ADC quantification was possible for 28 lesions with a diffusion restriction and clear, definable correlate on ADC maps. The median value of these lesions was 0.778 × 10^− 3^ mm^2^/s with an interquartile range of 0.723 to 0.912 × 10^− 3^ mm^2^/s. For a major proportion (56/84, 67%) of liver lesions, a clear delineation on ADC was not possible due to small lesion size and artifacts (e. g., subcapsular localisation resulting in susceptibility artifacts).

The median follow-up interval for combined ^18^F-DOPA-PET/CT and MRI was 484 days with an interquartile range of 250 to 769 days.

## Discussion

In the present study, we investigated the added diagnostic value of gadoxetic acid-enhanced MRI to ^18^F-DOPA-PET/CT for liver staging in patients with MTC. Complementary gadoxetic acid-enhanced MRI allowed for the detection of significantly more lesions compared to ^18^F-DOPA-PET/CT alone. In addition, liver lesion categorisation as benign or malignant was more conclusive at a significantly higher frequency, especially for small lesions with a diameter < 1 cm.

Our results are supported by a study that investigated ultrasound, CT, whole-body and liver MRI, and ^18^F-FDG-PET/CT for the detection of tumour recurrence and metastases in MTC patients with elevated serum calcitonin levels after initial treatment [32]. They found MRI to detect more liver metastases than the other imaging modalities, with MRI being the only modality to detect very small metastases (millimeter range) in two patients. The combination of MRI and CT detected significantly more metastases than ^18^F-FDG-PET/CT. However, the authors do not detail on the type of intravenous contrast agent and the comprehensive liver MRI protocol did not include DWI sequences. Our study extends the literature as it provides evidence for the use of multi-phase liver MRI with an intravenous, hepatobiliary contrast agent for liver staging in MTC including a late, hepatobiliary phase and diffusion-weighted images for the detection of liver metastases and for appropriate characterisation of liver lesions.

In line with our results, previous studies have shown a significant benefit of multi-phase contrast enhanced MRI for the detection of metastatic liver disease. In a head-to-head comparison of somatostatin receptor scintigraphy, multi-phase CT, and MRI in patients with neuroendocrine tumours for liver staging, MRI with intravenous Gd-DOTA administration allowed for the detection of significantly more metastases than CT and scintigraphy [33]. In addition, it was shown, that for the detection of liver metastases in patients with neuroendocrine tumours, the use of liver MRI using hepatobiliary contrast agent gadoxetic acid yields significant additional diagnostic value to nuclear imaging techniques [22]. Recent studies have also demonstrated that gadoxetic acid-enhanced MRI is superior for the detection of liver metastases to MRI with non-specific contrast agent or CT with contrast enhancement [29, 34]. In our cohort, DWI was an important co-feature for metastasis detection and characterisation. However, a significant proportion of MTC liver metastases showed a brisk arterial rim enhancement and a cystic centre, resulting in T2-shine through. As a consequence, our results suggest that DWI alone is not sufficient for liver metastasis detection and characterisation in MTC. Therefore, MTC liver staging should be performed based on all sequences of the comprehensive liver MRI protocol.

In our institution, ^18^F-DOPA-PET/CT is performed for whole-body tumour staging in MTC since numerous studies demonstrated superiority to ^18^F-FDG hybrid imaging [6, 8–10, 35]. The detection of liver metastases in MTC patients, however, is difficult due to the typically small size with correspondingly low ^18^F-DOPA uptake or size below the spatial resolution of PET imaging. Taken together, there is no existing consensus on imaging modalities for liver staging in patients with MTC. Our results strongly suggest that hepatobiliary contrast-enhanced liver MRI should be performed as routine part of whole-body tumour staging protocols in MTC. The hepatobiliary phase enabled by the use of the hepatocyte-specific contrast agent added significantly to the level of confidence in lesion characterisation. Future studies should further investigate the impact of multiphase liver MRI on clinical patient management, with regard to the various treatment options for metastatic liver disease as well as clinical endpoints such as progression-free and overall patient survival. Even in patients with multifocal liver metastases, the exact number and localisation of individual metastases may play a major role for therapy guidance, for instance to determine resectablility or eligibility for locoregional treatment options.

We acknowledge several limitations of the study. First, MRI was performed on two different 1.5 Tesla MRI scanners. Although imaging protocols were standardised, slight differences in image acquisition with potential impact on image reading cannot be fully excluded. Second, ^18^F-DOPA-PET/CT and gadoxetic acid enhanced MRI were performed within 30 days. Despite this relatively short interval, it is possible that patients with highly aggressive tumours experienced rapid tumor progression with new liver metastases between the two scans. However, the actual median time interval between gadoxetic acid-enhanced MRI and ^18^F-DOPA-PET/CT was 3 days (interquartile range 0–12 days), making short-term tumour progression unlikely. Third, a reading bias may be present since reading of one modality potentially influences the sensitivity of the reader for lesion detection on images of the other modality. To limit this bias, the reading order was randomised. Fourth, follow-up imaging was used as reference standard as no patient from the investigated population underwent liver biopsy. Fifth, the CT part of the ^18^F-DOPA-PET/CT scan was only performed in portal venous phase. Appending an unenhanced, an arterial, and a late dynamic phase may further improve the diagnostic performance of ^18^F-DOPA-PET/CT for the detection of (hypervascular) liver metastases. However, acquisition of additional CT phases increases radiation exposure and potentially requires a higher injection volume of iodinated contrast agent, which limits the applicability especially in young patients and chronic kidney disease.

## Conclusion

Gadoxetic acid-enhanced liver MRI significantly increases the detection rate for liver metastases in patients with MTC, particularly of small lesions with a diameter < 1 cm. In addition, gadoxetic acid-enhanced MRI allows for a definite lesion categorisation when ^18^F-DOPA-PET/CT remains inconclusive, with potential impact on clinical patient management and therapy guidance. Our results provide evidence for the routine use of gadoxetic acid-enhanced MRI as part of comprehensive staging protocols in MTC patients.

## Data Availability

The datasets that support the findings of this study are available from the corresponding author on reasonable request.

## Abbreviations

^18^F-DOPA: [18F]fluoro-dihydroxyphenylalanine
FOV: Field of view
GRE: Gradient echo
HASTE: Half fourier acquisition single shot turbo spin echo
MEN: Multiple endocrine neoplasia
MTC: Medullary thyroid carcinoma
TE: Time to echo
TR: Time to repetition
